# Large-Scale Drug Screening in Patient-Derived IDH^mut^ Glioma Stem Cells Identifies Several Efficient Drugs among FDA-Approved Antineoplastic Agents

**DOI:** 10.3390/cells9061389

**Published:** 2020-06-03

**Authors:** Philip Dao Trong, Gerhard Jungwirth, Tao Yu, Stefan Pusch, Andreas Unterberg, Christel Herold-Mende, Rolf Warta

**Affiliations:** 1 Department of Neurosurgery, Division of Experimental Neurosurgery, Heidelberg University Hospital, 69120 Heidelberg, Germany; Philip.DaoTrong@med.uni-heidelberg.de (P.D.T.); Gerhard.Jungwirth@med.uni-heidelberg.de (G.J.); Tao.Yu@med.uni-heidelberg.de (T.Y.); Andreas.Unterberg@med.uni-heidelberg.de (A.U.); H.Mende@med.uni-heidelberg.de (C.H.-M.); 2 Department of Neuropathology, Institute of Pathology, Heidelberg University Hospital, Clinical Cooperation Unit Neuropathology, German Cancer Research Center (DKFZ), 69120 Heidelberg, Germany; Stefan.Pusch@med.uni-heidelberg.de; 3 German Consortium of Translational Cancer Research (DKTK), 69120 Heidelberg, Germany

**Keywords:** drug screen, lower grade glioma, isocitrate dehydrogenase (IDH) mutant glioma stem cells, bortezomib, carfilzomib, daunorubicin, doxorubicin, epirubicin, omacetaxine, plicamycin

## Abstract

The discovery of the isocitrate dehydrogenase (IDH) mutation in glioma led to a paradigm shift on how we see glioma biology. Difficulties in cultivating IDH mutant glioma stem cells (IDH^mut^ GSCs) resulted in a paucity of preclinical models in IDH^mut^ glioma, limiting the discovery of new effective chemotherapeutic agents. To fill this gap, we used six recently developed patient-derived IDH^mut^ GSC lines and performed a large-scale drug screening with 147 Food and Drug Administration (FDA)-approved anticancer drugs. GSCs were subjected to the test compounds for 72 h in concentrations ranging from 0.0001 to 1 µM. Cell viability was assessed by CellTiterGlo and the induction of apoptosis by flow cytometry with Annexin V/propidium iodide staining. The initial screen was performed with two IDH^mut^ GSC lines and identified seven drugs (bortezomib, carfilzomib, daunorubicin, doxorubicin, epirubicin, omacetaxine, plicamycin) with a substantial antiproliferative activity, as reflected by half maximal inhibitory concentrations (IC_50_) below 1 µM and maximum inhibitory effects (E_max_) below 25%. These findings were validated in an additional four IDH^mut^ GSC lines. The candidate drugs, of which plicamycin and omacetaxine are known to cross the blood brain barrier, were used for subsequent cell death analyses. A significant induction of apoptosis was observed at IC_50_ values of the respective drugs. In summary, we were able to identify seven FDA-approved drugs that should be further taken into clinical investigations for the treatment of IDH^mut^ gliomas.

## 1. Introduction

The differentiation of glioma patients into isocitrate dehydrogenase (IDH) mutant and wildtype tumors (IDH^mut^/IDH^wt^) has proven to be a solid prognostic marker of patient outcome [1]. Patients harboring an IDH mutation fare much better than patients without [2]. This has led to a revision of the WHO classification in 2016, in which molecular parameters, such as the IDH mutation and 1p/19q co-deletion status, are now being incorporated along with classical histology [3]. Treatment consensus exists in IDH^wt^ glioma, such as glioblastoma (GBM), and comprises maximal safe resection, followed by adjuvant chemoradiation with temozolomide and the application of local tumor treating fields [4,5]. In IDH^mut^ lower grade glioma (LGG), the current standard is surgical resection, followed by procarbazine, lomustine, and vincristine (PCV) or temozolomide combined with radiation therapy. Compared to glioblastoma patients, treatment modalities and timing are not as well defined and depend on the patient’s risk stratification for recurrence and malignant transformation [6,7,8]. One reason for uncertainty is the paucity of preclinical models in IDH^mut^ glioma, which has hindered the development of innovative therapies. While IDH^wt^ glioma cell models have shown easy expansion and maintenance, difficulties in cultivating IDH^mut^ tumor cells have forced many to introduce the IDH mutation artificially into wildtype tumor cells, not accounting for the natural genetic background of the tumor [9,10]. Therefore, data generated with such models are unfortunately only of limited informative value. However, recently, we established several well-characterized IDH^mut^ glioma stem cell (GSC) cultures that could be used to gain further insight into the biology of LGG. These cultures grow as neurospheres, stably produce the oncometabolite (D)-2-hydroxyglutarate (D2HG), and have shown to induce tumor growth in xenograft models [11,12,13]. Its spherical growth enriches tumor stem cells, a subpopulation of tumor cells that are reminiscent of normal neural stem cells and drive tumor formation. Since they are thought to promote resistance to chemoradiation, it is crucial to a test potential chemotherapeutics on this subset of tumor cells [14,15,16]. One method to do so is to conduct a large-scale drug screening to discover effective and already approved chemotherapeutic agents without going through an extensive preclinical research period. This has already been shown to be feasible in patient-derived GBM GSCs [17,18,19]. 

In this study, we used a drug library consisting of 147 Food and Drug Administration (FDA)-approved drugs, tested pharmacologically-induced growth inhibition and induction of cytotoxic apoptosis in an initial screen with two IDH^mut^ GSC lines, and validated the results in four additional IDH^mut^ GSC lines. After going through a three-step filter based on parameters derived from the dose response curve (area under curve, half-maximal inhibitory concentration, maximum inhibitory effect), growth inhibitory and apoptotic inducing potential were then confirmed for the remaining seven candidate compounds, of which two have already been shown to cross the blood brain barrier (BBB). 

## 2. Materials and Methods

### 2.1. Cell Cultures and Drug Testing 

We used in total six IDH^mut^ glioma cell lines. Four of six of them were derived from IDH^mut^ gliomas classified as recurrent GBM WHO°IV, one patient harbored an anaplastic oligodendroglioma WHO°III and one patient was diagnosed with an anaplastic astrocytoma WHO°III (Table S1). GSC lines were cultivated as described before [20]. In brief, stem cells were grown as floating neurospheres in DMEM/F-12 medium, containing 20% BIT serum-free supplement, basic fibroblast growth factor (bFGF), and epidermal growth factor (EGF) at 20 ng/ml each (tumor stem cell (TSC) medium, all Provitro, Berlin, Germany). GSC lines were authenticated by short tandem repeat (STR) profiling (DMSZ, Braunschweig, Germany). The expression of several GSC markers, including CD133, SOX2, CD44, CSPG4, CD90, and nestin in the IDH^mut^ GSC lines, has been shown by Kohanbash et al. [12]. For drug testing, neurospheres were dissociated to single cells using accutase and plated at 8000 cells per well in a 96-well plate in TSC medium. The used drug library (AOD IX) was supplied by the NCI’s Developmental Therapeutics Program and consists of 147 FDA-approved cancer therapy agents (Table S1). For the initial screen, these were added to the IDH^mut^ GSC lines 24 h after cell plating at different concentrations (0.1 nM, 1 nM, 10 nM, 100 nM, 1000 nM). In order to increase the accuracy of the half maximal inhibitory concentration (IC_50_) values, the subsequent validation experiments were performed in a seven-step concentration range: 0.1 nM, 1 nM, 5 nM, 10 nM, 50 nM, 100 nM, and 1000 nM.

### 2.2. (D)-2-Hydroxyglutarate Measurements

D2HG levels were measured in the cell pellets of IDH^mut^ GSCs, as described by Balss et al. [21] In brief, (d)-2-hydroxyglutarate dehydrogenase catalyzes the reduction of NAD+ to NADH by oxidation of D2HG to α-ketoglutarate. NADH is then detected by a diaphorase/resazurin system measured by the fluorescent product, resorufin, which is exited at 540 nm and detected at 610 nm.

### 2.3. Cell Proliferation, Viability, and Apoptosis Assays

Cell proliferation was tested using the CellTiter-Glo^®^ 3D Cell Viability Assay (Promega, Madison, WI, USA), which measures ATP as an indicator of viability and generates a luminescent readout. The ready-to-use mix was added 72 h after compound inhibition, and luminescence was determined on a Tecan Infinite 200 reader (Tecan, Männedorf, Switzerland). Cell viability and induction of apoptosis was analyzed using Annexin V‒fluorescein isothiocyanate and propidium iodide staining. After drug treatment, neurospheres were centrifuged at 300 g at 4 °C, washed in 1 ml of ice-cold PBS, and gently dissociated into single cells using accutase. The washing step was then repeated three times. The GSCs were resuspended in 1 ml 1 x Annexin V binding buffer (Biolegend, San Diego, CA, USA), stained with 2 µl Annexin V antibody (Biolegend, San Diego, CA, USA), and incubated for 15 min in the dark at 4 °C. Finally, propidium iodide (PI) was added at a concentration of 50 µg/ml. Flow-cytometric measurements were done on a BD FACS-Calibur machine and analyzed with FlowJo X software (BD, Franklin Lakes, NJ, USA). 

### 2.4. Statistical Analyses

GraphPad Prism 7 (San Diego, CA, USA) was used to calculate dose response curves using normalized nonlinear regression with a sigmoid dose response to obtain the IC_50_, the area under the curve (AUC), and the E_max_. A student’s t-test was applied to test for differences in apoptosis between treated and control GSCs. Significance was considered if *p* < 0.05. 

## 3. Results

### 3.1. Composition of Drug Library

**Figure 1:**
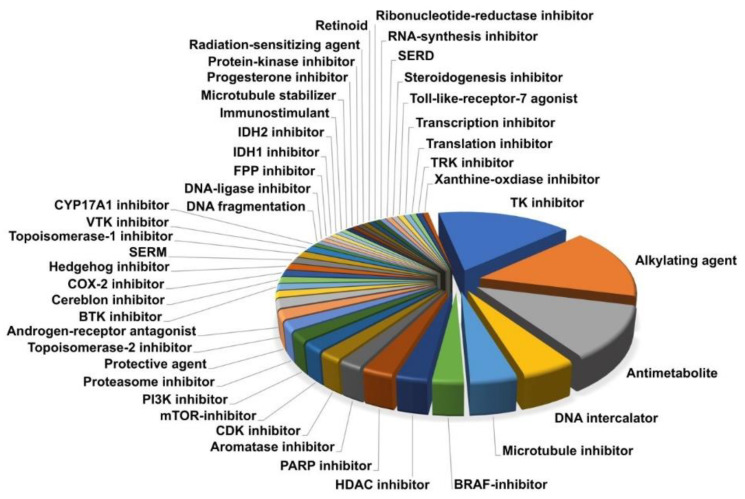
The drug library covers a broad spectrum of substance classes (*n* = 44), including a considerable range of anti-neoplastic modes of action used in modern cancer therapy.

In order to identify novel and effective antineoplastic drugs for the treatment of IDH^mut^ glioma, we performed a high-throughput screening, utilizing a drug library consisting of 147 drugs approved by the FDA for the treatment of different cancer types. These compounds covered a diverse spectrum of drug classes, mode of actions, and targets (Figure 1). The three main drug classes were tyrosine kinase inhibitors (blue, *n* = 24), alkylating agents (orange, *n* = 23), and antimetabolites (grey, *n* = 17). The remaining drugs were distributed over 41 subcategories, containing one to seven members and covering a considerable range of anti-neoplastic modes of action known in cancer therapy. Additionally, drugs currently being used in the treatment of IDH^mut^ glioma were included in the screening (temozolomide, procarbazine, lomustine, and vincristine). As many substances have potent antineoplastic activity in many cancer types, we tested all drugs irrespective of their ability to cross the BBB because many modern drug delivery systems, such as convection enhanced delivery (CED), may overcome this obstacle. Detailed information on all drugs of the screened library can be found in Table S2.

### 3.2. Drug Screening in IDH^mut^ Glioma Stem Cells Identifies Seven FDA-Approved Drugs

To identify promising drugs for the treatment of IDH^mut^ glioma, we used two IDH^mut^ GSC lines for the initial screen derived from an astrocytoma WHO°III (NCH1681) and a recurrent GBM WHO°IV (rGBM, NCH551b). These have already been extensively used in other studies [11,12,13,22,23,24]. All cells were maintained as spheroid cultures in stem cell media, thus enriching tumorigenic stem cells and allowing them to produce significant levels of the oncometabolite D2HG by the mutant IDH enzyme (Table S1).

**Figure 2:**
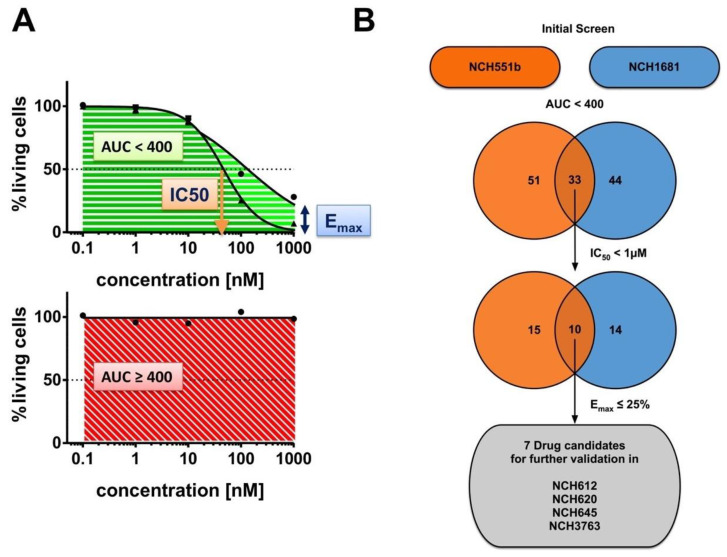
Drug screening workflow. A total of 147 Food and Drug Administration (FDA)-approved drugs were tested in two isocitrate dehydrogenase (IDH) mutant (IDH^mut^) glioma stem cell (GSC) lines (NCH551b, NCH1681) in a 5-dose primary screen. (**A**) The following drug response parameters were analyzed: area under the curve (AUC), half-maximal inhibitory concentration (IC_50_), and maximal inhibitory effect (E_max_). (**B**) Drugs which first showed an AUC < 400 (*n* = 33) and secondly an IC_50_ < 1 µM (*n* = 10) in both cell lines were then ultimately filtered for an E_max_ ≤ 25%. Candidate drugs were successfully tested in four additional IDH^mut^ GSC lines (NCH612, NCH620, NCH645, NCH3763) in a 7-dose secondary validation.

To primarily identify drugs that are effective at low concentrations with IC_50_ values in the nM range, we tested five concentrations starting from 0.1 nM to 1000 nM. Cell growth was assessed by the CellTiterGlo 3D assay. We analyzed several dose-response parameters, such as the area under the curve (AUC), the half maximal inhibitory concentration (IC_50_), and the maximum inhibitory effect (E_max_) (Figure 2A). Each parameter by itself could not assess the overall characteristics of a drug. For example, a drug that had a low IC_50_ but a high E_max_ might not be as potent as a drug with a higher IC_50_ but an E_max_ at zero (and thus a high AUC). Therefore, we applied a three-step filter process to select drugs for the subsequent validation (Figure 2B).

**Figure 3:**
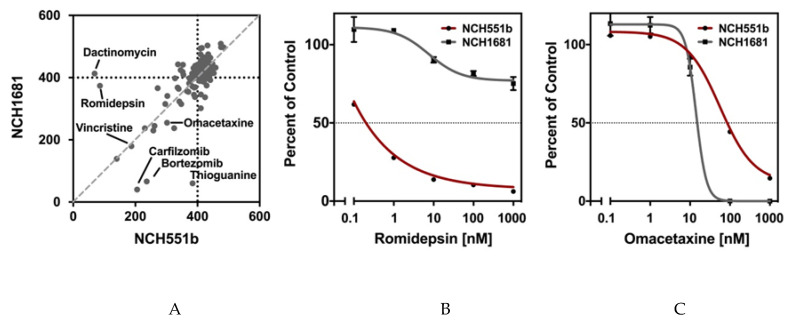
NCH551b and NCH1681 were chosen for the initial drug screen. (**A**) Most of the drugs clustered around an AUC of 400m suggesting little to no growth inhibition. (**B**) Some compounds showed an inhibition in only one of the cell lines (e.g., romidepsin NCH551b). (**C**) Compounds were considered for further analyses when growth inhibition was observed in both cell lines (e.g., omacetaxine).

We first compared the AUCs of NCH551b and NCH1681 for all tested drugs. As expected, we observed that most drugs had little to no effect on cell growth, even at the highest concentrations tested (Figure 3A). This is shown by the cluster of dots with an AUC around 400, the value which indicates no cell growth inhibition and represents a cell survival of 100% in all five concentrations. However, there were also highly effective drugs marked by a low AUC. Interestingly, some drugs affected only one of the GSC lines (e.g., thioguanine, dactinomycin, romidepsin), while others inhibited the growth of both GSC lines to the same degree (e.g., omacetaxine) (Figure 3B,C and Table S3). Nevertheless, we identified 62 drugs (42.2%) inducing growth inhibition in either or both GSC lines (NCH551b: *n* = 51, 34.7%; NCH1681: *n* = 44, 29.9%). Since we aimed to identify drugs for the treatment of IDH^mut^ glioma regardless of the WHO grade, only the drugs demonstrating comparable effects in both GSC lines were further considered. After using strict selection criteria of AUC below 400, IC_50_ below 1 µM, and E_max_ less than or equal 25%, the following seven candidate drugs remained: daunorubicin, doxorubicin, epirubicin, bortezomib, carfilzomib, plicamycin, and omacetaxine. Interestingly, of the established chemotherapies, PCV, and temozolomide, only vincristine showed growth inhibition in both GSC lines (Figure 3A and Figure S1). However, it did not withstand our criterion of an E_max_ ≤ 25% (Table S3). 

### 3.3. Validation of Growth Inhibititory Effects of Candidate Drugs in Four Additional IDH^mut^ GSCs 

**Figure 4:**
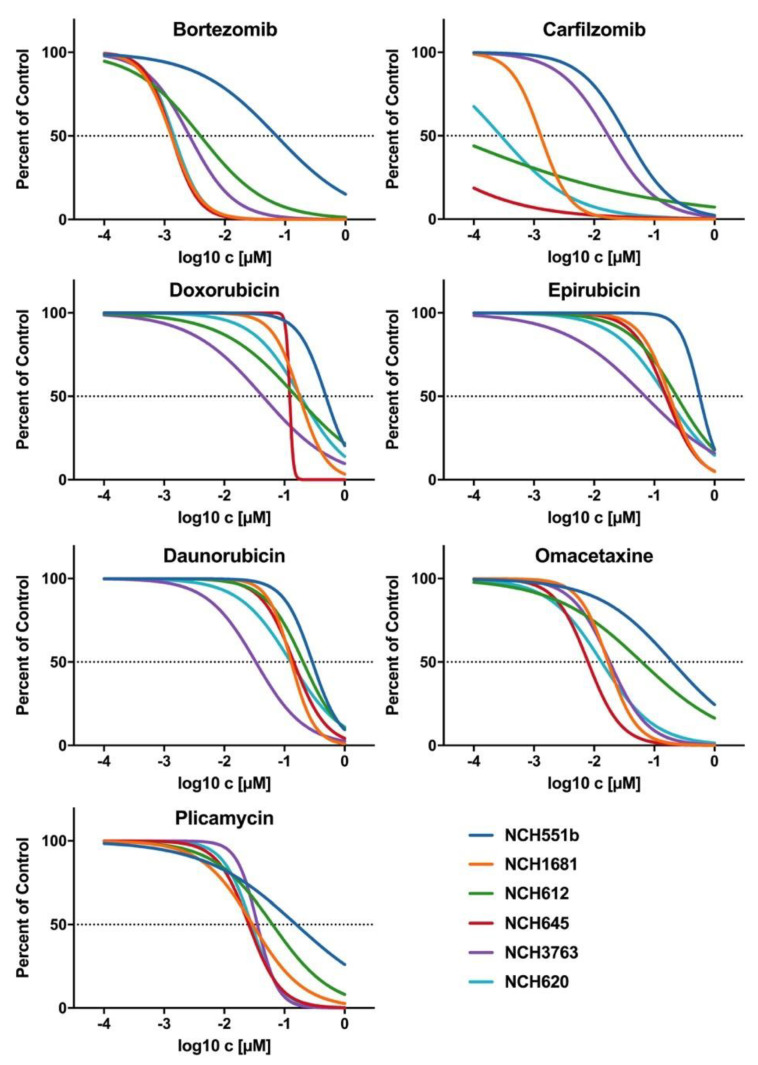
Representative dose response curves of candidate drugs for all six IDH^mut^ GSC lines. The proteasome inhibitors, bortezomib and carfilzomib, showed the biggest variance in IC_50_, whereas the anthracylines (doxorubicin, daunorubicin, and epirubicin), as well plicamycin and omacetaxine, showed a range of IC_50_ within a power of 10. All candidate drugs had an IC_50_ < 1µM and a high maximum inhibitory effect of E_max_ ≤ 17%.

**Figure 5:**
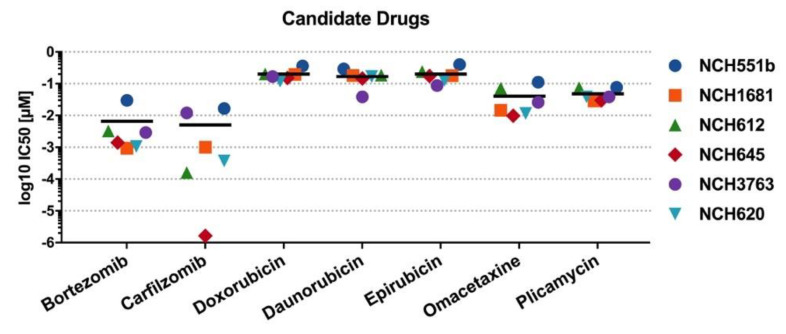
IC_50_ values of candidate drugs. Mean IC_50_ is marked by a horizontal line. Carfilzomib had the broadest range of drug sensitivity and had the greatest effect in NCH645 (red diamond), with an IC_50_ below the lowest tested concentration of 0.1 nM.

To corroborate our findings of the initial drug screening, the candidate drugs were tested on four additional IDH^mut^ GSC lines (rGBM WHO°IV: NCH620, NCH645, NCH3763; oligodendroglioma WHO°III: NCH612). Furthermore, dose-response testing with a total of seven concentrations, ranging from 0.1 nM to 1000 nM, was refined to improve the accuracy of the IC_50_ values. Representative dose-response curves of one biological replicate are shown for all seven drugs in Figure 4. All seven drugs induced comparable growth inhibition in the four additional GSC lines, as seen in NCH551b and NCH1681. However, a considerable range of IC_50_ values depending on the substance class between the GSC lines was observed. Bortezomib and carfilzomib, both proteasome inhibitors, showed the lowest IC_50_ values of all validated drugs (mean: 6.6 nM ± 11.4 nM and 5.0 nM ± 7.3 nM, respectively). Interestingly, they were also the drugs with the broadest IC_50_ ranges (0.94–29.8 nM; 1.64 pM–17.5 nM, respectively, Figure 5, Table 1). 

Each of the remaining five compounds fell within a fairly narrow range. Mean IC_50_ values of plicamycin and omacetaxine, as well as daunorubicin, doxorubicin, and epirubicin, exceeded proteasome inhibitors by a factor of 10 or 40, respectively (Table 1, Figure 5). Taken together, validation of the candidate drugs in additional GSC lines confirmed our primary screening results. Next, we selected one representative drug from every drug class for further investigations. Therefore, carfilzomib (second generation proteasome inhibitor), doxorubicin (anthracycline), omacetaxine (reversible protein-synthesis inhibitor), and plicamycin (RNA synthesis inhibitor) were used for subsequent cell death analyses.

### 3.4. Cell Death Analysis by Annexin V and Propidium Iodide 

**Figure 6:**
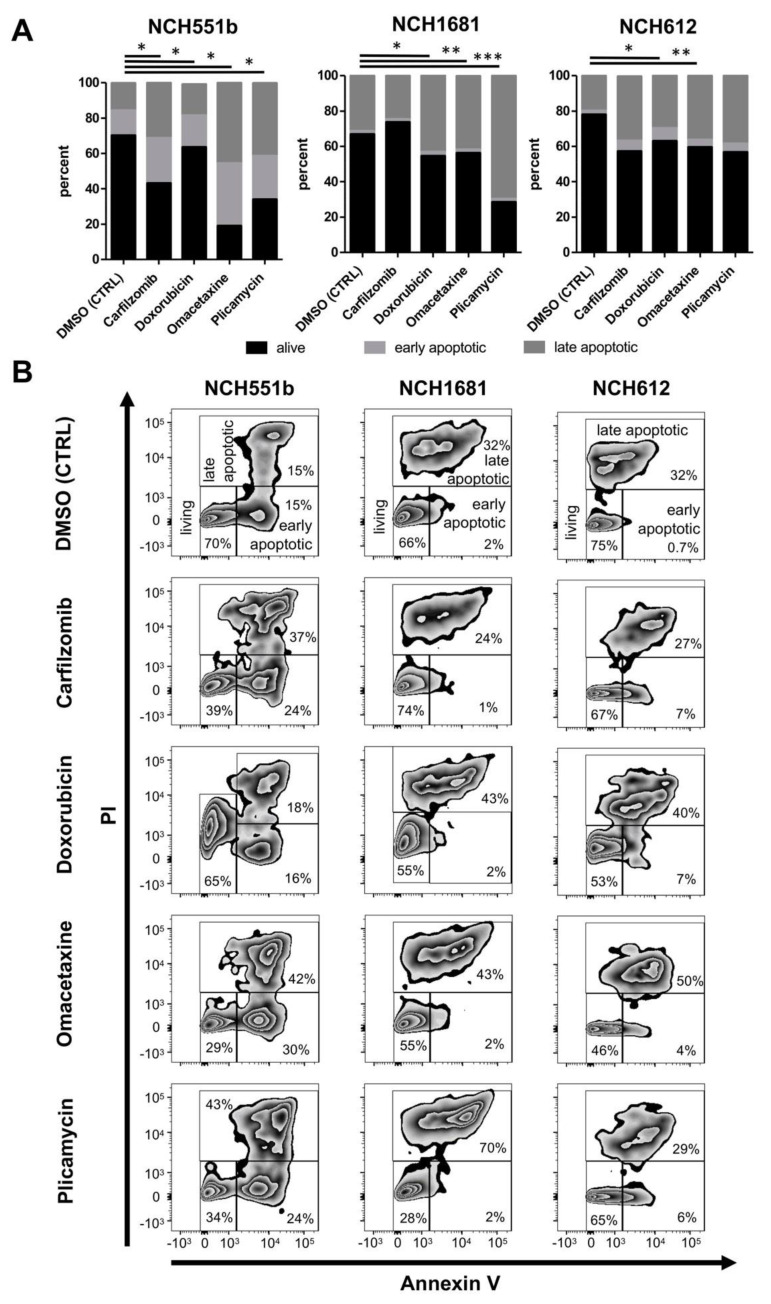
Cell apoptosis measured by Annexin V/PI flow cytometry for NCH551b, NCH1681, and NCH612 for the candidate drugs at the IC_50_ concentrations established in the previous experiments for each GSC line. (**A**) Percentage of living/apoptotic cells (mean) (**B**) Representative flow cytometry plots. Fluorescent measurements confirmed a significantly elevated number of cells undergoing apoptosis compared to the control. (Students t-test) * *p* < 0.05; ** *p* < 0.01, *** *p* < 0.001; propidium iodide (PI).

To further corroborate the effectiveness of the aforementioned drugs, induction of apoptosis was determined by using Annexin V/PI staining. Carfilzomib, doxorubicin, omacetaxine, and plicamycin were tested in NCH551b, NCH1681, and NCH612 GSC lines at the previously determined IC_50_ concentrations ni NCH551b, NCH1681 and NCH612 (Figure 6). The three GSC lines represent three different histological entities (rGBM, anaplastic astrocytoma, oligodendroglioma) and were chosen to elaborate if differences in the amount of apoptosis could be detected according to an IDH mutant glioma subtype. Cell death analyses via Annexin / PI staining enabled us to distinguish three different states of the cells (Figure 6). (1) Unaffected living cells were PI and Annexin V negative (living). (2) Living early apoptotic cells were characterized by an intact cell membrane and expression of phosphatidylserine, which was detected by Annexin V (early apoptotic; PI negative but Annexin V positive). (3) In contrast, late apoptotic cells featured a leaky membrane, which resulted in an influx of PI, together with binding of Annexin V (late apoptotic; PI positive and Annexin V positive). After 72 h of treatment, Annexin V/PI stainings confirmed a significantly elevated number of cells undergoing apoptosis, compared to the DMSO control in most of the cell lines (Figure 6). NCH551b showed the most pronounced apoptotic effect for all tested drugs, followed by NCH1681 and NCH612. The most dramatic reduction of living cells from 70% to 19% was seen in NCH551b after treatment with omacetaxine (*p* < 0.05). In addition, in NCH551b, both early and late apoptotic fractions increased, as compared to NCH1681 and NCH612, where mostly the late apoptotic fraction increased. In NCH1681 and NCH612, the apoptotic effect was less pronounced as in NCH551b, reaching statistical significance in 3/4 and 2/4 drugs, respectively. As NCH612 is a slow proliferating oligodendroglial GSC line, one could speculate, if that might be the reason for the differences seen compared to the faster growing cell lines NCH551b and NCH1681. Taken together, we conclude that the candidate drugs not only showed growth inhibition in the CellTiterGlo measurements but were also capable of inducing apoptosis at the IC_50_ concentrations established in our previous experiments. 

In summary, we screened 147 FDA-approved drugs using six IDH^mut^ GSC lines and identified seven drugs that effectively reduced GSC growth at very low concentrations. Furthermore, we have demonstrated that four representative drugs were able to induce cell death in three IDH^mut^ GSC lines, including two highly effective candidates, which are known to cross the BBB (omacetaxine, plicamycin) [25,26].

## 4. Discussion

In this study, we used a library of 147 FDA-approved anticancer drugs to screen for cytotoxic activity in six IDH^mut^ GSC lines. Seven drugs, of which two have already shown BBB penetration (omacetaxine and plicamycin), have shown a potent antiproliferative effect in the initial screen, already at low concentrations in the well-characterized tumorigenic GSC lines, NCH551b and NCH1681 [25,26]. These findings could be confirmed for all candidate drugs in the additional four IDH^mut^ GSC lines. Subsequent cell death analysis by Annexin V and PI flow cytometry confirmed effective induction of apoptosis and significantly diminished living cells at the previously determined IC_50_ values. In summary, our analysis revealed seven promising drug candidates for the treatment of IDH^mut^ glioma, which warrants further preclinical and clinical testing. 

Despite the less aggressive nature of low-grade IDH^mut^ glioma, most of the patients ultimately end up with a malignantly progressed tumor with very limited treatment options [1,2]. To date, treatment regimens consist of maximal safe resection, followed by either a watch and wait strategy or subsequent chemoradiation, depending on the risk profile of the individual patient [27,28]. PCV (procarbazine, lomustine, vincristine) or temozolomide, combined with radiation therapy, have made their way into clinical routines [6,7,8]. Both chemotherapeutic regimens have been chosen based on sparse preclinical data, mostly relying on extrapolation from studies of high-grade glioma not yet stratified for IDH mutation status [7,27]. Therefore, we aimed to discover new effective chemotherapeutic agents by screening 147 FDA-approved cancer drugs for cytotoxic activity. To our knowledge, this is the first study conducting a systematical identification process for novel but approved chemotherapeutic agents by using GSC lines with an endogenous IDH mutation. Among the 147 drugs tested for growth inhibition, we were able to identify seven highly effective drug candidates (bortezomib, carfilzomib, daunorubicin, doxorubicin, epirubicin, omacetaxine, and plicamycin). In addition, the flow-cytometric measurements of Annexin V/PI-stained GSCs confirmed that the drugs not only inhibit cell growth, as measured by CellTiterGlo, but also effectively induce cell death.

Interestingly, several of the candidate compounds have also been identified in previous drug screenings, conducted with either glioma cell lines or other tumor cell lines, such as neuroblastoma or renal cell carcinoma (bortezomib [29,30], carfilzomib [31], omacetaxine [32,33,34], plicamycin [35]). This suggests that these compounds may have modes of action that are at least partially independent of the tumor entity. If the previously described unique tumor biology of IDH-mutant glioma results in a higher sensitivity against our candidate drugs, it remains to be answered by further investigations. 

Of the seven candidate drugs identified in the initial screen, plicamycin (aka mithramycin) and omacetaxine (aka homoharringtonin), were the only drugs where penetration of the BBB has been reported [25,26,36,37]. Plicamycin, an antineoplastic antibiotic produced by *Streptomyces plicatus*, is an RNA synthesis inhibitor [38]. Our results demonstrate that plicamycin has a strong anti-tumor activity in GSC lines. It was tested in clinical studies in the late 1960s in a small number of malignant glioma patients and was approved for malignant testicular cancer in 1970 [36,39]. Although it showed little effect on the survival of malignant glioma patients, it is questionable if these results can be translated into cancer therapy after recent revision of the WHO classification. Additionally, newer plicamycin derivatives with even better antitumor activity and less toxicity make this compound attractive for modern glioma treatment [40,41]. Omacetaxine, a reversible protein-synthesis inhibitor, has been approved by the FDA for chronic- or accelerated-phase chronic myeloid leukemia (CML) patients who develop intolerance to two or more tyrosine-kinase inhibitors [42]. In our study, omacetaxine showed robust growth inhibition in all six IDH^mut^ GSC models and was a potent inducer of apoptosis. One study tested omacetaxine in recurrent malignant glioma patients in a phase II study, with little clinical benefit [43]. This may be due to the fact that the study cohort consisted of 14 recurrent glioblastomas and only one recurrent anaplastic glioma patient—not accounting for IDH status therefore was not translatable to our experimental setup. 

Bortezomib and carfilzomib are proteasome inhibitors and showed the strongest inhibitory effect at doses below the lowest tested concentration of 0.1 nM (carfilzomib, NCH645). Their antineoplastic effect is based on the inhibition of the proteasome. This leads to an accumulation of ubiquitinated misfolded proteins and subsequently induces apoptosis [44]. Bortezomib showed a potent in vitro cytotoxic activity in several malignant glioma studies but failed to show in vivo efficacy when given intravenously, most likely because of its inability cross the BBB [45,46]. Carfilzomib has shown to inhibit proliferation, migration, and invasion in vitro in GBM stem cells, although in vivo studies are lacking so far [47]. Doxorubicin, daunorubicin, and epirubicin belong to the substance class of anthracyclines, with a wide spectrum of use in oncology [48]. Anthracyclines are not able to cross the BBB, but since they have also shown a good antineoplastic activity in glioma studies, they are being extensively tested with new drug delivery systems, using, e.g., liposomes, nanoparticles, convection-enhanced delivery, and focused ultrasound [49,50,51,52]. Furthermore, local intraoperative treatments, coupled with a sustained release drug delivery system, have shown promising results in an orthotopic murine model with two human IDH^mut^ glioma and one chondrosarcoma cell lines [53]. 

Of the existing chemotherapeutic agents already being administered to IDH^mut^ glioma patients (procarbazine, lomustine, vincristine, and temozolomide), only vincristine showed growth inhibition in our initial drug screen in both cell lines. Although it did not pass our criterion of E_max_ ≤ 25%, this finding does support the validity of our preclinical model.

Since the tested drugs have already successfully received FDA-approval, we believe that our in vitro results may enable faster translation into in vivo studies and subsequent clinical trials. Several projects performing large drug screenings are underway, sharing the same motive [54]. However, they mostly rely on widely-used long-term cultured adherent cell lines and include only IDH^wt^ glioma cell lines. This limits the usability of their results for the treatment of IDH^mut^ glioma, since glioma stem cells grown as neurospheres seem to have a distinct drug sensitivity compared to cells grown adherently [55]. Furthermore, cells grown as neurospheres mimic the parental tumor more closely. This is demonstrated by the fact that GSC lines with an endogenous IDH mutation and a maintained 2HG production can be solely grown as sphere cultures [13,56]. Hence, we used a total of six low-passaged patient-derived IDH^mut^ neurosphere cultured GSC lines and proved a robust inhibition of proliferation for all identified candidate drugs at nanomolar concentrations. Despite known difficulties in establishing IDH^mut^ GSCs, our GSC panel included one astrocytoma WHO°III and one oligodendroglioma WHO°III, in addition to the four rGBM stem cell lines. We do acknowledge that minor differences in drug sensitivity between the GSC cultures exist and that the limited number of GSC lines per tumor subtype does not allow us to draw conclusions as to associations of tumor subtypes to a specific substance class. These differences may be caused by intertumoral heterogeneity, as described in IDH^wt^ glioblastoma [34]. Personalized treatment strategies are thought to overcome this issue. However, the number of druggable mutations and genomic aberrations in IDH^mut^ glioma is relatively low [57]. Only the IDH mutation seems to be a highly conserved feature, making it an attractive target. Unfortunately, promising results of IDH^mut^ inhibitors in lymphatic cancers and in preclinical models of IDH^mut^ glioma did not translate into clinical effectiveness for the treatment of lower-grade glioma [58]. Therefore, there is still an urgent need for the development of therapies applicable to IDH^mut^ glioma patients.

## 5. Conclusions

In this study, we used six unique patient-derived IDH^mut^ glioma stem cell lines to screen 147 FDA-approved drugs for antineoplastic and apoptotic activity. We were able to identify seven highly effective compounds demonstrating potent activity in all GSC lines. As new drug delivery systems, such as local applications, are constantly evolving, not only the drugs with a proven BBB penetration (omacetaxine and plicamycin) represent attractive candidates, but also the proteasome inhibitors (bortezomib, carfilzomib) and anthracyclines (doxorubicin, epirubicin) are worth considering for future preclinical and clinical testing. 

## Figures and Tables

**Table 1:** Data obtained for candidate drugs on six IDH^mut^ GSC lines.

Drug	Parameter	NCH551b	NCH1681	NCH612	NCH645	NCH3763	NCH620	Mean	SD
Bortezomib	IC_50_ [nM]	29.8	0.9	3.3	1.4	2.9	1.1	6.6	11.4
	E_max_ [%]	24.2	3.2	4.1	1.6	5.5	1.1	6.6	8.8
Carfilzomib	IC_50_ [nM]	16.5	1.0	0.2	<0.1	12.1	0.4	5.0	7.3
	E_max_ [%]	15.9	3.2	17.0	2.1	0.9	0.0	6.5	7.8
Doxorubicin	IC_50_ [nM]	361.3	196.0	202.1	152.4	167.7	120.6	200.0	84.4
	E_max_ [%]	20.6	0.0	0.0	0.0	18.6	9.3	8.1	9.6
Daunorubicin	IC_50_ [nM]	290.5	181.2	187.1	145.2	38.7	167.0	168.3	81.0
	E_max_ [%]	11.6	0.0	0.0	0.0	16.2	0.0	4.6	7.3
Epirubicin	IC_50_ [nM]	399.4	178.3	241.7	173.7	87.2	125.9	201.0	110.4
	E_max_ [%]	18.1	0.0	0.0	0.0	24.8	7.5	8.4	10.7
Omacetaxine	IC_50_ [nM]	110.8	14.6	71.9	9.8	26.1	11.6	40.8	41.4
	E_max_ [%]	23.6	1.2	0.0	0.0	14.7	0.7	6.7	10.1
Plicamycin	IC_50_ [nM]	76.8	28.8	73.6	29.9	38.5	38.7	47.7	21.7
	E_max_ [%]	37.2	13.2	25.3	3.6	8.0	11.5	16.5	12.5

Half-maximal inhibitory concentration (IC_50_), maximum inhibitory effect (E_max_), standard deviation (SD).

## Supplementary Materials

The following are available online at https://www.mdpi.com/2073-4409/9/6/1389/s1, Figure S1: Exemplary dose response curves of classical treatments, Table S1: Patient and cell line characteristics, Table S2: Drug library used for screening, Table S3: Drug screening results of 147 FDA-approved drugs in NCH551b and NCH1681.

## Abbreviations

IDH	Isocitrate Dehydrogenase
mut	mutant
wt	wildtype
FDA	Food and Drug Administration
LGG	Lower grade glioma
HGG	Higher grade glioma
GBM	Glioblastoma
PCV	Procarbazine, lomustine, Vincristine
GSC	Glioma stem cell
DTP	Developmental Therapeutics Program
NCI	National Cancer Institute
bFGF	Basic fibroblast growth factor
EGF	Epidermal growth factor
TSC	Tumor stem cell
STR	Short tandem repeat
D2HG	(D)-2-hydroxyglutarate
IC_50_	Half-maximal inhibitory concentration
AUC	Area under the curve
E_max_	Maximum inhibitory effect
SD	Standard deviation
PI	Propidium iodide
BBB	Blood brain barrier
