# Supplementary figures and images for: Large-Scale Drug Screening in Patient-Derived IDHmut Glioma Stem Cells Identifies Several Efficient Drugs among FDA-Approved Antineoplastic Agents
Source: Cells. 2020 Jun 3;9(6):1389. doi: 10.3390/cells9061389 (PMC7348988; doi:10.3390/cells9061389)

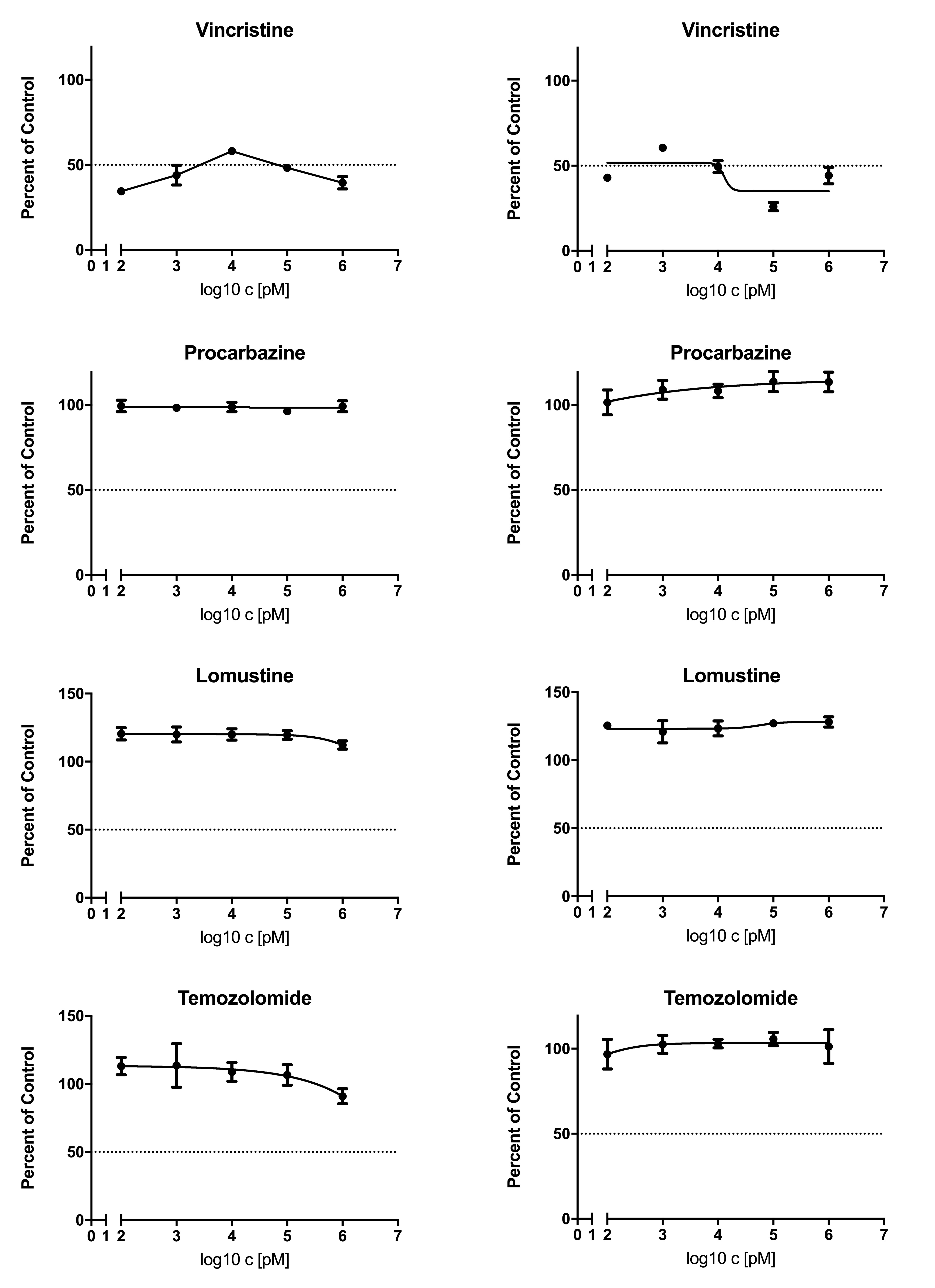

Supplement: Supplementary file 1 [file cells-09-01389-s001.zip › Figure S1.jpg]
